# Comparison of Ropivacaine and Bupivacaine in Intraperitoneal Instillation for Postoperative Analgesia in Laparoscopic Cholecystectomy: A Randomized Controlled Trial

**DOI:** 10.7759/cureus.73167

**Published:** 2024-11-06

**Authors:** Shahbaz Hasnain, Raavi S Lakshmi Priya

**Affiliations:** 1 Anaesthesiology, Dr. D Y Patil Medical College, Hospital and Research Centre, Dr. D Y Patil Vidyapeeth, Pune, IND

**Keywords:** bupivacaine, intra-peritoneal analgesia, laparoscopic cholecystectomy, postoperative pain, rescue analgesia, ropivacaine, visual analogue scale

## Abstract

Introduction

Laparoscopic cholecystectomy, despite its several advantages, is sometimes associated with discomfort due to pain in the immediate postoperative period. Effective management of this pain is critical for enhancing recovery, minimizing complications, and facilitating early discharge. The use of local anesthetics for intra-abdominal analgesia, specifically bupivacaine and ropivacaine, has been investigated as a means to improve postoperative pain control. This study aims to compare the analgesic efficacy of intra-peritoneal ropivacaine versus bupivacaine following laparoscopic cholecystectomy.

Methods

This prospective, randomized, comparative study was conducted after obtaining institutional ethics committee clearance. Sixty ASA grade I/II patients, aged 18 to 65 years, undergoing elective laparoscopic cholecystectomy were enrolled. Patients were randomly assigned to receive either 35 ml of 0.375% ropivacaine (Group R) or 35 ml of 0.25% bupivacaine (Group B) administered intra-peritoneally. Analgesic efficacy was assessed using Visual Analogue Scale (VAS) scores, heart rate monitoring, and the requirement for rescue analgesia. Data were collected at various time intervals and analyzed using statistical methods.

Results

Mean heart rate values were significantly lower in Group R compared to Group B from the 2nd to 8th hour and at the 18th hour postoperatively (p < 0.05). The mean VAS scores for pain at rest were significantly lower in Group R compared to Group B from the 8th to 24th hour (p < 0.001). A substantially higher proportion of patients in Group B required rescue analgesia compared to Group R (p < 0.05). Specifically, 16.7% of patients in Group R required rescue analgesia versus 43.3% in Group B. The time to the first dose of rescue analgesia was longer in Group R (8.4±3.6 hours) compared to Group B (6.8±11.3 hours), though this difference was not statistically significant (p > 0.05). Total analgesic consumption was significantly higher in Group B (20.5±3.2 mg) compared to Group R (14.6±2.8 mg) (p < 0.001).

Conclusion

Ropivacaine provides superior postoperative analgesia compared to bupivacaine when used for intra-peritoneal instillation in laparoscopic cholecystectomy. The findings support the use of ropivacaine for effective pain management in this surgical context, potentially leading to improved patient outcomes and reduced opioid consumption.

## Introduction

Laparoscopic cholecystectomy has emerged as the preferred approach for gallbladder removal procedures due to its minimally invasive procedure and associated advantages over open surgery [1]. Laparoscopic cholecystectomy, a minimally invasive surgical procedure for the removal of the gallbladder, has become the gold standard treatment for symptomatic cholelithiasis (gallstones) and other gallbladder-related disorders. Although laparoscopic surgeries are associated with reduced postoperative pain, minimized hospital stay, and faster recovery compared to open procedures, patients often experience significant discomfort and pain in the early postoperative period. Effective management of postoperative pain is crucial not only for patient comfort but also for reducing the risk of complications, enhancing recovery, and facilitating early discharge compared to open cholecystectomies; these operations result in a smoother recovery and reduced pain following surgery [2]. Patients typically experience visceral discomfort following an operative laparoscopy, but parietal pain (abdominal wall) is the predominant side effect of laparotomy [2,3]. After laparoscopic cholecystectomy, shoulder pain resulting from diaphragmatic irritation due to CO2 pneumoperitoneum is a commonly observed postoperative occurrence [4].

Following laparoscopic cholecystectomy, abdominal pain may arise due to distension of the parietal peritoneum from intraperitoneal gas insufflation, the secretion of inflammatory pain mediators, or irritation caused by residual blood [5]. Intraperitoneal instillation of local anesthetics has gained popularity due to its potential to provide effective analgesia by blocking the nociceptive pathways involved in visceral and parietal pain. Bupivacaine and ropivacaine are two widely used long-acting amide local anesthetics that have been studied for intraperitoneal instillation in laparoscopic cholecystectomy [6,7]. While both agents have demonstrated analgesic efficacy, there is ongoing debate regarding their relative potency, duration of action, and potential for adverse effects, particularly when administered intraperitoneally. One such long-acting local anesthetic that doesn't cause nausea or vomiting, gastritis from nonsteroidal anti-inflammatory drugs [NSAIDs], or drug dependence from opioids is bupivacaine [8]. Recently, ropivacaine has become available as a long-acting local anesthetic. It works longer than bupivacaine and has less toxicity and adverse effects. Comparing it to Bupivacaine, it likewise has a favorable cardiovascular safety record [6].

Numerous interventional studies have explored the concurrent use of general anesthesia with intraperitoneal and port-site local anesthetic administration during laparoscopic cholecystectomy [7,9-11]. Approximately half of these investigations demonstrated a significant reduction in postoperative discomfort. These studies evaluated various commonly used local anesthetic agents at different concentrations.

Therefore, we conducted this randomized prospective clinical study to compare the effects of intraperitoneal instillation of bupivacaine or ropivacaine to relieve postoperative pain following laparoscopic cholecystectomy.

## Materials and methods

Study design 

This study was designed as a prospective, randomized, and comparative investigation. Before the commencement of this study, ethical approval was obtained from the Institutional Ethics Committee (IESC/PGS/2022/153) and the clinical trial registry of India under the registration number CTRI/2023/06/053604. The study involved 60 patients, aged 18 to 65 years, classified as ASA (American Society of Anesthesiologists) grade I/II, of either gender, who met all inclusion and exclusion criteria and were scheduled for elective laparoscopic cholecystectomy.

Sample size

The sample size was calculated using “WINPEPI” software (11.3) with the following specifications: The study was based on a study conducted by Babu et al. [12]. Comparing the effectiveness of intraperitoneal instillation of ropivacaine versus bupivacaine for postoperative pain relief following laparoscopic cholecystectomy. Considering the visual analog score mean (standard deviation) in group R was 4.573 (0.379) and in group B was 4.185 (0.354), level of significance of 1% and power as 90%, and assuming 10% attrition due to patient dropout, the sample size calculated was 60, with 30 in each group. We included 60 patients in this study who were randomized into two groups.

Patient selection

60 patients undergoing laparoscopic cholecystectomy under general anesthesia were randomly assigned into 2 groups of 30 patients each, designated as Group R and Group B, following the obtaining of informed written consent from the patients in their understandable language. Computer-generated randomization was used to assign the patients to one of the groups. Thirty participants in Group B received 35 mL of 0.25% Bupivacaine, and thirty participants in Group R received 35 mL of 0.375% Ropivacaine.

Inclusion and exclusion criteria

The study included ASA grade I or II patients aged between 18 and 65 years of either sex who provided written informed consent and were scheduled for laparoscopic cholecystectomy under general anesthesia. Additionally, participants were required to be hemodynamically stable with routine investigations within normal limits. Exclusion criteria were ASA physical status III or higher, patients outside the 18-65 age range, those undergoing emergency procedures, and patients with major neurological, cardiac, respiratory, metabolic, renal, or hepatic diseases, coagulation abnormalities, or known allergies to the study drug.

Preoperative assessment and preparation

On the day before surgery, all patients underwent a thorough evaluation, including a detailed medical history and general physical and systemic examination. Routine laboratory investigations were conducted, and all patients were kept nil per oral (NPO) for at least 6 hours before surgery. In the preoperative room, patient vital signs such as pulse rate, blood pressure, respiratory rate, oxygen saturation, and ECG were recorded while they rested in a supine position. Intravenous access was established using a 20G cannula.

Anesthesia protocol

Patients received general anesthesia following a standardized protocol. Pre-medication included an injection of glycopyrrolate (0.004 mg/kg) and midazolam (0.02 mg/kg). Patients were pre-oxygenated with 100% oxygen for 3 minutes. Induction agents included fentanyl (2 mcg/kg) and propofol (2 mg/kg). Once mask ventilation was confirmed, succinylcholine (2 mg/kg) was administered to facilitate intubation. After intubation, a long-acting muscle relaxant, vecuronium (0.1 mg/kg), was given. Anesthesia was maintained with sevoflurane and air throughout the procedure. As part of the institutional protocol, paracetamol (1 g) was administered intraoperatively for analgesia.

Drug administration

At the final stage of surgery, before removing the trocars, intraperitoneal drug instillation was performed by the surgeon. In both groups, 35 ml of the assigned drug (0.25% Bupivacaine or 0.375% Ropivacaine) was divided and instilled into three regions: 15 ml in the hepatic diaphragmatic space, 10 ml in the gallbladder bed, and 10 ml between the liver and kidney. At the end of the surgery, patients were reversed with neostigmine (0.05 mg/kg) and glycopyrrolate (0.008 mg/kg) before extubation [13,14]. The patients in this study were blinded to the treatment they received.

Postoperative pain assessment and management

Postoperative pain was assessed using a visual analog scale (VAS) at multiple time points: immediately after surgery (T0), hourly for the first 4 hours (T1), every 4 hours up to 12 hours (T2), and every 6 hours up to 24 hours (T3). Rescue analgesia, consisting of intravenous tramadol (50 mg), was provided if the VAS score exceeded 3. Additional doses were administered if the VAS score reached 5 or higher. The timing and frequency of rescue analgesia were meticulously recorded.

Statistical analysis

The collected data were entered into Microsoft Excel 2016 and analyzed using statistical software IBM Corp. Released 2021. IBM SPSS Statistics for Windows, Version 28.0. Armonk, NY: IBM Corp. Categorical variables were expressed as frequencies and percentages, while continuous variables were reported as mean (standard deviation). An independent t-test and multiple ANOVA were used to compare differences between groups, with a p-value of <0.05 considered statistically significant.

## Results

The mean age in Group R (39.7 ± 10.6 years) was slightly lower than in Group B (43.2 ± 10.5 years), but the difference was not statistically significant (p-value = 0.205). In the study, Group R consisted of 30 participants, with nine males (30%) and 21 females (70%), while Group B also had 30 participants, with 12 males (40%) and 18 females (60%).

A two-way repeated-measures ANOVA was conducted to examine the effects of time, treatment (Ropivacaine vs. Bupivacaine), and their interaction on the heart rate. The analysis assumed sphericity and was conducted with an alpha level of 0.05. Results indicated a significant main effect of time, F=7.898, p<0.0001, suggesting significant changes in the heart rate over time. There was no significant interaction between time and treatment (F = 1.488, p=0.1696, indicating that changes over time did not significantly differ between Ropivacaine and Bupivacaine treatments. We further sought to analyze the change in heart rate at each time point between the two groups. At baseline, there was no significant difference between Group R (97.8 ± 12.7) and Group B (94.96 ± 13.9) with a p-value of 0.448. By the 2nd hour, Group B showed significantly higher values (80.5 ± 9.6) compared to Group R (74.6 ± 9.7) with a p-value of 0.02. This trend of higher values in Group B continued at the 3rd, 4th, 8th, and 18th hours, all with statistically significant differences (p-values of 0.002, 0.04, 0.02, and <0.05, respectively). At the 12th and 24th hours, the differences were not significant, with p-values of 0.1 and 0.85, respectively (Table 1). 

**Table 1:** Mean heart rate (per minute) in the two groups at different intervals of time (hours). Each group had 30 participants. A t-test was used to compare the two groups; p  < 0.05 was considered statistically significant.

Time (h)	Group R (Mean±SD)	Group B (Mean±SD)	p-value
Baseline	97.8±12.7	94.96±13.9	0.448
1^st^ hour	84.8±11.57	87.6±10.06	0.31
2^nd^ hour	74.6±9.7	80.5±9.6	0.02
3^rd^ hour	79±9.5	85.5±5.8	0.002
4^th^ hour	80.7±10.9	86.2±10.04	0.04
8^th^ hour	79.8±9.5	85.5±8.8	0.02
12^th^ hour	79.5±9.5	83.2±7.6	0.1
18^th^ hour	77.6±8.3	82.8±5.6	<0.05
24^th^ hour	78.2±9.1	77.8±9.3	0.85

The Visual Analogue Scale (VAS) scores were compared between Group R and Group B over time (Table 2). A two-way repeated-measures ANOVA was conducted to examine the effects of time, treatment (Ropivacaine vs. Bupivacaine), and their interaction on the pain at rest (VAS). The analysis assumed sphericity with an alpha level of 0.05. Results indicated a highly significant main effect of time, F = 96.20, P<0.0001. Further, to analyze the effect at each time point, we performed a T-test. At the 1st hour, Group R had a mean VAS score of 2.1 ± 0.5, while Group B had a slightly higher mean of 2.4 ± 0.78, with a p-value of 0.08, indicating no significant difference. Similarly, at the 2nd, 3rd, and 4th hours, the VAS scores showed no significant differences between the two groups (p-values of 0.32, 0.62, and 0.46, respectively). However, by the 8th hour, Group B (3.6 ± 0.62) had a significantly higher VAS score compared to Group R (3.2 ± 0.83), with a p-value of 0.04. This significant difference continued at the 12th (3.5 ± 0.56 for Group R vs. 3.8 ± 0.59 for Group B, p = 0.04), 18th (3.2 ± 0.76 for Group R vs. 3.6 ± 0.74 for Group B, p = 0.04), and 24th hours (2.7 ± 0.41 for Group R vs. 2.9 ± 0.24 for Group B, p = 0.02), indicating that Group B experienced higher pain levels during these time points.

**Table 2:** Pain at rest (VAS) in the two groups at different intervals of time (hours). Each group had 30 participants. VAS = visual analog scale. A t-test was used to compare two groups; p < 0.05 was considered statistically significant.

Time (h)	Group R (Mean ± SD)	Group B (Mean ± SD)	p-value
1st hour	2.1 ± 0.5	2.4 ± 0.78	0.08
2nd hour	2.5 ± 0.79	2.7 ± 0.78	0.32
3rd hour	2.6 ± 0.8	2.7 ± 0.78	0.62
4th hour	2.7 ± 1.02	2.9 ± 1.09	0.46
8th hour	3.2 ± 0.83	3.6 ± 0.62	0.04
12th hour	3.5 ± 0.56	3.8 ± 0.59	0.04
18th hour	3.2 ± 0.76	3.6 ± 0.74	0.04
24th hour	2.7 ± 0.41	2.9 ± 0.24	0.02

In terms of the requirement for rescue analgesia, 25 participants in Group R (83.3%) did not require additional pain relief, compared to 17 participants in Group B (56.7%). Conversely, 5 participants in Group R (16.7%) required rescue analgesia, while 13 participants in Group B (43.3%) needed it. Both groups had a total of 30 participants each, and the difference was statistically significant. This suggests that participants in Group R had a lower requirement for rescue analgesia compared to those in Group B (Table 3).

**Table 3:** Requirement of rescue analgesia in the two groups. Chi-square test was used.

Requirement of rescue analgesia	Group R (n=30)	Group B (n=30)	p-value
No	25 (83.3%)	17 (56.7%)	0.02
Yes	5 (16.7%)	13 (43.3%)	
Total	30 (100%)	30 (100%)	

In Group R, 25 participants (83.3%) did not require any rescue analgesia, while 5 participants (16.7%) needed it once. In Group B, 17 participants (56.7%) did not require rescue analgesia, and 13 participants (43.3%) needed it once. The difference between the two groups was statistically significant, with a p-value of 0.02 (Table 4).

**Table 4:** Comparison of the number of times rescue analgesia is required in study groups. A chi-square test was used.

number of times rescue analgesia required	Group R (n=30)	Group B (n=30)	p-value
0	25 (83.3%)	17 (56.7%)	0.02
1	5 (16.7%)	13 (43.3%)	
Total	30 (100%)	30 (100%)	

The time to the first dose of rescue analgesia was similar between the groups, with Group R having a mean of 8.4 ± 3.6 hours and Group B having a mean of 6.8 ± 11.3 hours, with a p-value of 0.46, indicating no significant difference. However, the total analgesic consumption was significantly higher in Group B, with a mean of 20.5 ± 3.2 mg compared to Group R, which had a mean of 14.6 ± 2.8 mg (Table 5).

**Table 5:** Comparison of time to 1st dose of rescue analgesia and total analgesia consumption among groups A t-test was used to compare the two groups.

Variables	Group R (n=30)	Group B (n=30)	p-value
Time to 1^st^ dose of rescue analgesia(hrs)	8.4±3.6	6.8±11.3	0.46
Total analgesia consumption(mg)	14.6±2.8	20.5±3.2	<0.001

## Discussion

The present prospective, randomized study evaluated the efficacy and safety of intraperitoneal instillation of ropivacaine versus bupivacaine for postoperative analgesia following laparoscopic cholecystectomy. Our findings revealed that ropivacaine was associated with a significant reduction in postoperative pain scores at rest compared to bupivacaine, especially during the early postoperative period.

The study conducted by Babu et al. concluded that both ropivacaine and bupivacaine are effective for postoperative pain relief, with ropivacaine potentially offering a better cardiovascular safety profile. Babu et al. used 20 ml of 0.2% ropivacaine and 20 ml of 0.25% bupivacaine, finding no significant difference in efficacy between the two anesthetics [12]. However, their study suggested that ropivacaine might be a preferable alternative due to its favorable safety profile.

In contrast, our study utilized higher volumes and concentrations: 35 ml of 0.375% ropivacaine and 35 ml of 0.25% bupivacaine. This approach resulted in improved analgesic effects with ropivacaine compared to bupivacaine. The increased concentration and volume of ropivacaine used in our study likely contributed to its superior analgesic efficacy. This difference highlights that while lower doses of ropivacaine and bupivacaine may show comparable results, higher doses of ropivacaine can provide enhanced pain relief, making it a potentially more effective choice for managing postoperative pain.

Heart rates were significantly lower in Group R compared to Group B for a prolonged period, which can be attributed to the more dense and prolonged analgesia provided by ropivacaine. This difference in heart rate was statistically significant from the 2nd to the 8th hour and again at the 18th hour. This finding aligns with the results of Sharan R et al., who also observed significant heart rate differences between groups receiving local anesthetics at similar time points [13].

Our results demonstrated that ropivacaine led to significantly lower pain scores from the 8th to the 12th hour post-surgery. This finding is consistent with the literature, where ropivacaine has been shown to offer prolonged analgesia due to its pharmacological properties, including lower lipophilicity and higher selectivity for sensory nerve fibers compared to bupivacaine [14]. These properties likely contribute to the reduced pain scores observed in our study, as ropivacaine's lower propensity for motor blockade facilitates better patient comfort and early mobilization [15]. In contrast to our study, Rademaker et al. did not find a significant decrease in postoperative discomfort between the ropivacaine and bupivacaine groups [16]. Rademaker et al. attributed their lack of success to the relatively minimal dosage of local anesthetics used and a smaller sample size, which may have limited their ability to detect significant differences in pain relief. Our findings are consistent with the results of a study by Meena et al., which demonstrated that ropivacaine provided better pain relief compared to bupivacaine, especially from the fifth to the twelfth postoperative hour [17]. Similarly, Rapolu et al. reported that ropivacaine was associated with lower pain scores and reduced opioid consumption compared to bupivacaine in various surgical contexts [18]. Shivhare et al. also found that ropivacaine provided effective analgesia with a reduced incidence of motor blockade compared to bupivacaine [19]. Our results are further supported by several other studies showing that ropivacaine provides better pain relief postoperatively [6,20]. 

In our study, the number of patients requiring rescue analgesia was significantly higher in the bupivacaine group compared to the ropivacaine group. Specifically, rescue analgesia was administered to patients in the bupivacaine group starting from the 6th hour, whereas in the ropivacaine group, it was required less frequently, primarily at the 8th and 12th hours. This finding is consistent with the results of Meena et al., who reported that patients receiving ropivacaine required less total analgesia compared to those receiving bupivacaine [17]. 

Significance statement

The findings of the study indicate that Ropivacaine provided significantly lower Visual Analog Scale (VAS) scores compared to Bupivacaine at various time points postoperatively. While the statistical significance (p < 0.05) demonstrates a reliable difference between the two groups. The reduction in VAS scores with Ropivacaine, particularly noted from the 8th hour onward, represents a clinically meaningful decrease in postoperative pain. The statistical significance of the reduced VAS scores with Ropivacaine is noteworthy; the clinical significance lies in its potential to enhance patient comfort, reduce reliance on additional analgesics, improve overall satisfaction, and promote better recovery trajectories. These factors highlight the importance of choosing appropriate analgesic regimens in postoperative care and aligning clinical practices with evidence-based findings for optimal patient outcomes.

Limitations

This study has several limitations that should be acknowledged. Firstly, the findings are based on a single-center study, and multi-center studies are needed to validate these results across diverse settings. This would help account for variations in surgical techniques, anesthesia protocols, and patient demographics, providing a robust and generalized assessment. Additionally, there may be methodological limitations, such as potential biases in patient selection and randomization procedures, which could impact the internal validity and reliability of the results. Furthermore, a sample size of 30 in each group might be underpowered to detect subtle differences, especially in secondary outcomes like rescue analgesia timings. The study primarily focused on pain scores and opioid consumption as primary outcomes, which, while important, represent only a part of the overall impact of analgesic interventions. Incorporating additional patient-reported outcomes, such as quality of life, functional recovery, and satisfaction with anesthesia, would offer a more comprehensive evaluation of the clinical impact of these anesthetics. Addressing these limitations in future research could enhance the robustness and applicability of the findings.

## Conclusions

This prospective, randomized study compared the efficacy of intraperitoneal instillation of ropivacaine versus bupivacaine for postoperative pain management following laparoscopic cholecystectomy. The results showed that ropivacaine provided significantly better pain relief, with lower VAS scores from the 8th to 24th hour post-surgery. Patients in the ropivacaine group also had lower heart rates and a reduced need for rescue analgesia compared to the bupivacaine group. Additionally, total analgesic consumption was significantly lower in the ropivacaine group. These findings suggest that ropivacaine offers superior analgesia and may reduce opioid use, enhancing recovery. However, further multi-center studies with larger sample sizes must confirm these results and assess broader clinical applicability.
